# Supplementary material for: High diversity of mosquito vectors in Cambodian primary schools and consequences for arbovirus transmission
Source: PLoS One. 2020 Jun 5;15(6):e0233669. doi: 10.1371/journal.pone.0233669 (PMC7274438; doi:10.1371/journal.pone.0233669)
Supplement: S1 File — (DOCX) [file pone.0233669.s002.docx]

**S2 Supporting Information.**

**Results of generalized linear mixed model (GLMM) using Template Model Builder (TMB) using Family Poisson with ‘school’ as a random effect reflecting the random choice of the schools on all mosquitoes, all vector mosquitoes, Japanese encephalitis virus vectors, Dengue virus vectors and *Plasmodium spp.* Vectors.**

1. **All Mosquitoes**

| AIC | BIC | Log Lik | Deviance | Df residual |
| --- | --- | --- | --- | --- |
| 19835.4 | 19866.2 | -9905.7 | 19811.4 | 84 |

Random effects:

| GroupsName | Variance | Std.Dev. |
| --- | --- | --- |
| school(Intercept) | 0.2437 | 0.4936 |

Number of obs: 96, groups: school, 24

| ALL MOSQUITOES | Estimate | Std. Error | z value | Pr(>\|z\|) |
| --- | --- | --- | --- | --- |
| (Intercept) | 5.02E+00 | 7.08E+00 | 0.71 | 0.48 |
| dvlptrural | 2.95E-01 | 3.91E+00 | 0.08 | 0.94 |
| dvlpturban | 9.53E-01 | 7.77E+00 | 0.12 | 0.9 |
| pagodapagoda | 2.02E-02 | 3.51E+00 | 0.01 | 1 |
| school.area | -1.59E-05 | 3.77E-05 | -0.42 | 0.67 |
| nb.classroom | 4.76E-02 | 1.43E+00 | 0.03 | 0.97 |
| nb.students | 1.41E-03 | 3.01E-02 | 0.05 | 0.96 |
| pop.village | -2.65E-04 | 1.98E-03 | -0.13 | 0.89 |
| monthFebruary | -3.85E-02 | 2.09E-01 | -0.18 | 0.85 |
| monthMay | -3.75E-01 | 2.38E-01 | -1.58 | 0.11 |
| monthNovember | -2.18E-01 | 2.19E-01 | -0.99 | 0.32 |

1. **All vectors**

| AIC | BIC | Log Lik | Deviance | Df residual |
| --- | --- | --- | --- | --- |
| 17480.8 | 17511.6 | -8728.4 | 17456.8 | 84 |

Random effects:

| GroupsName | Variance | Std.Dev. |
| --- | --- | --- |
| school(Intercept) | 0.2778 | 0.5271 |

Number of obs: 96, groups: school, 24

| ALL VECTORS | Estimate | Std. Error | z value | Pr(>\|z\|) |
| --- | --- | --- | --- | --- |
| (Intercept) | 4.38E+00 | 7.07E+00 | 0.62 | 0.536 |
| dvlptrural | 5.95E-01 | 3.91E+00 | 0.15 | 0.879 |
| dvlpturban | 1.07E+00 | 7.76E+00 | 0.14 | 0.89 |
| pagodapagoda | -3.72E-03 | 3.50E+00 | 0 | 0.999 |
| school.area | -3.05E-06 | 3.70E-05 | -0.08 | 0.934 |
| nb.classroom | 2.94E-02 | 1.43E+00 | 0.02 | 0.984 |
| nb.students | 2.33E-03 | 3.00E-02 | 0.08 | 0.938 |
| pop.village | -3.32E-04 | 1.97E-03 | -0.17 | 0.866 |
| monthFebruary | -4.55E-02 | 2.19E-01 | -0.21 | 0.836 |
| monthMay | -6.54E-01 | 2.75E-01 | -2.38 | 0.017 * |
| monthNovember | -2.81E-01 | 2.34E-01 | -1.2 | 0.23 |

1. **JEV vectors**

| AIC | BIC | Log Lik | Deviance | Df residual |
| --- | --- | --- | --- | --- |
| 17126.3 | 17157.0 | -8551.1 | 17102.3 | 84 |

Random effects:

| GroupsName | Variance | Std.Dev. |
| --- | --- | --- |
| school(Intercept) | 0.2775 | 0.5268 |

Number of obs: 96, groups: school, 24

| JEV vectors | Estimate | Std. Error | z value | Pr(>\|z\|) |
| --- | --- | --- | --- | --- |
| (Intercept) | 4.20E+00 | 7.02E+00 | 0.6 | 0.5497 |
| dvlptrural | 7.06E-01 | 3.91E+00 | 0.18 | 0.8566 |
| dvlpturban | 1.20E+00 | 7.85E+00 | 0.15 | 0.8789 |
| pagodapagoda | -2.88E-02 | 3.60E+00 | -0.01 | 0.9936 |
| school.area | 7.48E-06 | 3.63E-04 | 0.02 | 0.9836 |
| nb.classroom | 1.41E-02 | 1.54E+00 | 0.01 | 0.9927 |
| nb.students | 2.55E-03 | 3.07E-02 | 0.08 | 0.9338 |
| pop.village | -3.21E-04 | 1.96E-03 | -0.16 | 0.8699 |
| monthFebruary | -4.40E-03 | 2.23E-01 | -0.02 | 0.9842 |
| monthMay | -7.26E-01 | 2.76E-01 | -2.64 | 0.0084 ** |
| monthNovember | -2.69E-01 | 2.39E-01 | -1.13 | 0.2601 |

1. **DENV vectors**

| AIC | BIC | Log Lik | Deviance | Df residual |
| --- | --- | --- | --- | --- |
| 787.7 | 818.4 | -381.8 | 763.7 | 84 |

Random effects:

| GroupsName | Variance | Std.Dev. |
| --- | --- | --- |
| school(Intercept) | 0.2196 | 0.4686 |

Number of obs: 96, groups: school, 24

| DENV vectors | Estimate | Std. Error | z value | Pr(>\|z\|) |
| --- | --- | --- | --- | --- |
| (Intercept) | 1.58E+00 | 8.81E-01 | 1.79 | 0.073 |
| dvlptrural | 3.40E-01 | 4.87E-01 | 0.7 | 0.485 |
| dvlpturban | -8.22E-01 | 9.91E-01 | -0.83 | 0.407 |
| pagodapagoda | 1.66E-01 | 4.47E-01 | 0.37 | 0.711 |
| school.area | -4.27E-05 | 4.51E-05 | -0.95 | 0.343 |
| nb.classroom | 9.70E-02 | 1.92E-01 | 0.5 | 0.614 |
| nb.students | 1.81E-04 | 3.85E-03 | 0.05 | 0.963 |
| pop.village | 9.05E-05 | 2.42E-04 | 0.37 | 0.708 |
| monthFebruary | -4.71E-01 | 1.43E-01 | -3.28 | 0.001 ** |
| monthMay | 4.96E-01 | 1.13E-01 | 4.4 | 1.1E-05 *** |
| monthNovember | -1.13E+00 | 1.80E-01 | -6.29 | 3.3E-10 *** |

1. **Plasmodium vectors**

| AIC | BIC | Log Lik | Deviance | Df residual |
| --- | --- | --- | --- | --- |
| 699.9 | 730.7 | -338.0 | 675.9 | 84 |

Random effects:

| GroupsName | Variance | Std.Dev. |
| --- | --- | --- |
| school(Intercept) | 0.8846 | 0.9405 |

Number of obs: 96, groups: school, 24

| Plasmodium vectors | Estimate | Std. Error | z value | Pr(>\|z\|) |
| --- | --- | --- | --- | --- |
| (Intercept) | 2.06E+00 | 2.07E+00 | 1 | 0.32 |
| dvlptrural | -1.14E+00 | 1.18E+00 | -0.96 | 0.34 |
| dvlpturban | -8.40E-02 | 2.31E+00 | -0.04 | 0.97 |
| pagodapagoda | -5.97E-01 | 1.08E+00 | -0.55 | 0.58 |
| school.area | 6.81E-05 | 1.04E-04 | 0.66 | 0.51 |
| nb.classroom | -1.20E-01 | 4.55E-01 | -0.26 | 0.79 |
| nb.students | 1.09E-03 | 9.22E-03 | 0.12 | 0.91 |
| pop.village | -2.40E-05 | 5.84E-04 | -0.04 | 0.97 |
| monthFebruary | -8.12E-02 | 3.23E-01 | -0.25 | 0.8 |
| monthMay | -3.02E-01 | 3.43E-01 | -0.88 | 0.38 |
| monthNovember | 4.32E-02 | 3.13E-01 | 0.14 | 0.89 |
