# Supplementary material for: High diversity of mosquito vectors in Cambodian primary schools and consequences for arbovirus transmission
Source: PLoS One. 2020 Jun 5;15(6):e0233669. doi: 10.1371/journal.pone.0233669 (PMC7274438; doi:10.1371/journal.pone.0233669)
Supplement: Attachment — Submitted filename: PLOS ONE ReviewsSM.docx [file pone.0233669.s004.docx]

PLOS ONE. **ONE-D-19-20103 R1: High diversity of mosquito vectors in Cambodian primary schools and consequences for arbovirus transmission**

# ANSWERS TO REVIEWERS

**Reviewer #3**

Authors have collected mosquitoes in school and have observed high diversity. Some of mosquitoes collected are known to be vectors. However, authors don't report any evidence of presence of parasite or virus in mosquitoes collected but have largely discussed the transmission risk in schools. Since it is possible (not too difficult) to detect parasites (e.g. Plasmodium spp) and virus (e.g. DENV and other) in mosquitoes, I strongly suggest to authors to pool mosquitoes collected per species per school in order to search parasites and virus in these mosquitoes. Then, authors would be more consistent with their conclusion.

We agree with the reviewer comments about virus/parasites detection in the mosquitoes collected. This is mainly a financial issue. We hope to have more projects in the future to be able to detect what is really circulating. Even if the entomological surveillance is difficult, with an estimation of less than 1 positive mosquito on 1,000 mosquitoes estimated, even during outbreak (few examples exist: Rift Valley fever in Madagascar, Zika in French Guyana…).

One sentence was added in the conclusion line 265: “To be exhaustive, a screening of pathogens carried by mosquitoes in schools should be implemented”.

**Reviewer #4**

Overall, this manuscript is very important in providing the mosquito diversity in the country especially because authors mention that there are few data available on mosquito diversity in the country. In understanding that it might be referenced a lot in the future once published - it is important to make sure the manuscript is well written, the data analysis is appropriately and well performed, and the recommendations for vector control options are very clearer.

Anyway, authors have attempted to the large extent to address reviewers' comments. However, more work still need to be done, especially on the data analysis and recommendations of what needs and can be done.

Given that there are so many random effects – I would have expected the analysis to be done using a generalized linear mixed model (GLMM) which takes into account random effects in addition to the usual fixed effects rather than using GLM. The statistician should check the analysis and the numbers presented. It might be the case that the characterization of the landscape is very difficult and takes time but incorporation of seasonality shouldn't take time.

Thank you for this remark. The reviewer was right: We indeed go back to the data by using glmm (function glmmTMB) in R, and observe significant effects, even if slight when looking at the data.

In fact, we have randomly selected our schools at the beginning, without taking into account the representativeness of all the schools in the region studied according to different parameters (number of students, pop dependent on this school, primary / college, urban / rural,% patients with the virus in the study area (or 5 km away, etc.). This is why we considered that the school is random. In fact, by putting the school in random effect, the GLMM calculated a regression for each school and the coefficients estimated for the other variables without the school bias.

At the end of the analysis, we repeat a shapiro.test on the residues, and we found a better result with residuals closer to normal. We also test the overdistention (surdispersion) of data and corrected it.

To summary, we finally did a glm analysis by taking schools as random effect.

The other random effect could have be the month of capture because, for organizational reasons, we did samplings every 3 months. So we also tested the month as a random factor. But there was no difference in the quality of the model (almost the same AIC) and we chose to include the month in the variables to be able to study its effect.

For the other point, as the observational study is on only one year, we are not able to study the seasonality as there is no replicates in time. We hope to be able to work on a several years’ observation database.

We precised in the text (Material and Method part, Statistical Analysis section) which analysis we did. We changed all this part. Regarding the results, we added the statistical effect in the text. Moreover we add a Supplementary file for the results of the analysis.

This manuscript could have been strong if the study also looked at the breeding sites in school surroundings and its correlations with mosquitoes caught in the classroom.

We also agree with that. We observed few breeding sites (in toilets schools, and few plastic garbage) for looking more specifically at Dengue vectors, but we did not do a complete characterization of all breeding sites in 24 schools (logistic and financial reasons). Also, this would have been another full study.

Authors should provide a clear recommendations of the vector control options and their justification. The options should be discussed in the discussion section stating why they are expected to work, how they will be implemented? etc? Especially in such a setting (i.e., classroom - school environment)

We remained global regarding recommendations for vector control because we did not study or characterize the insecticide resistance of the most dominant species, furthermore, we did not study their biology including the breeding sites as mentioned earlier.
